# Combination of histochemical analyses and micro-MRI reveals regional changes of the murine cervix in preparation for labor

**DOI:** 10.1038/s41598-021-84036-9

**Published:** 2021-03-01

**Authors:** Antara Chatterjee, Rojan Saghian, Anna Dorogin, Lindsay S. Cahill, John G. Sled, Stephen Lye, Oksana Shynlova

**Affiliations:** 1 Physiology, University of Toronto, Toronto, Canada; 2 Sinai Health System, Lunenfeld-Tanenbaum Research Institute, Toronto, ON Canada; 3 Medical Biophysics, University of Toronto, Toronto, Canada; 4 Mouse Imaging Centre, Hospital for Sick Children, Toronto, Canada; 5 Obstetrics and Gynecology, University of Toronto, Toronto, Canada

**Keywords:** Reproductive biology, Magnetic resonance imaging

## Abstract

The cervix is responsible for maintaining pregnancy, and its timely remodeling is essential for the proper delivery of a baby. Cervical insufficiency, or “weakness”, may lead to preterm birth, which causes infant morbidities and mortalities worldwide. We used a mouse model of pregnancy and term labor, to examine the cervical structure by histology (Masson Trichome and Picrosirius Red staining), immunohistochemistry (Hyaluronic Acid Binding Protein/HABP), and ex-vivo MRI (T_2_-weighted and diffusion tensor imaging), focusing on two regions of the cervix (i.e., endocervix and ectocervix). Our results show that mouse endocervix has a higher proportion of smooth muscle cells and collagen fibers per area, with more compact tissue structure, than the ectocervix. With advanced gestation, endocervical changes, indicative of impending delivery, are manifested in fewer smooth muscle cells, expansion of the extracellular space, and lower presence of collagen fibers. MRI detected three distinctive zones in pregnant mouse endocervix: (1) inner collagenous layer, (2) middle circular muscular layer, and (3) outer longitudinal muscular layer. Diffusion MRI images detected changes in tissue organization as gestation progressed suggesting the potential application of this technique to non-invasively monitor cervical changes that precede the onset of labor in women at risk for preterm delivery.

## Introduction

Lasting for about 40 weeks, human pregnancy requires an intimate physiological interaction between mother and fetus. At term, the preparedness of the uterus (i.e., uterine smooth muscle, myometrium, and cervix) is crucial for successful delivery of a baby^1–3^. Premature opening of the cervix has been implicated in preterm birth (PTB), the delivery of a neonate prior to 37 weeks of gestation^4,5^. PTB affects 10.6% of pregnancies worldwide causing significant infant mortality and morbidities, and rates are continuing to increase^6–8^. Current methods to predict PTB are inadequate, which is due to the multifactorial etiology of this disease and significant gaps in our understanding of the physiological, biochemical and structural changes in the reproductive tract that lead to PTB. Multiple lines of evidence point to changes in the cervix as common factor that lead to delivery, both at term and preterm. For instance, during a rapid process of cervical remodeling prior to delivery, called “ripening”, the human cervix undergoes a remarkable transformation from a rigid closed structure to a completely compliant organ^9–13.^ It is unclear, however, whether a premature cervical ripening leads to PTB in cases of cervical insufficiency (CI), or a “weak cervix”^14–18^. Currently the gold standard for detecting CI is the shortening of cervical length (CL) to less than 25 mm in pregnant women under 24 weeks of gestation, as measured by transvaginal ultrasonography (TVUS)^14^. However, studies using TVUS to measure CL and time to delivery show variable results, as some women exhibit gradual changes in cervical shortening while others undergo accelerated cervical shortening^19^. These inter-individual variations complicate the ability of TVUS measurements of CL to predict PTB. This method has a low positive predictive value (27%^5^), indicating that changes in CL only cannot reliably forecast the upcoming PTB. Magnetic resonance imaging (MRI) is a non-invasive technology with the potential to reveal more subtle effects of cervical ripening by detecting structural changes in the cervical stroma, such as water content and tissue rigidity^20^, in addition to detecting changes in cervical dimensions^21–23^. MRI protocols can be sensitized to specific features of cellular structure such as the restrictions on water diffusion thereby aiding in a thorough examination of microstructure^24^. This technology could elucidate a sequence of cervical events that occur in preparation for labor onset, which may improve PTB prediction in high-risk pregnant women.

Anatomically, the non-pregnant cervix in women and mice is a small, yet firm, cylinder with a canal inside surrounded by region-specific cervical lumen^25^, separated from the uterus and vagina by two physical openings, the internal os and external os, with well-defined endocervix and ectocervix sub-regions^26–28^. The cervix is designated the role of “the gatekeeper of pregnancy”, as the internal os/endocervix acts as a structural barrier to increasing pressure by the growing fetus, while the external os/ectocervix acts as protective barrier to infections ascending from the vagina^29^. Structurally, cervix is made up from extracellular matrix (ECM), consisting mostly from elastin and collagen fibers (70% collagen type I with 30% collagen type III), and glycosaminoglycans (e.g. hyaluronic acid/HA), as well as cellular components (smooth muscle cells/SMCs, fibroblasts, vascular cells, luminal epithelium and resident immune cells)^13,^^30–32^. Proportionally, the cervix has more fibrous tissue, than the body of the uterus. The predominance of collagen fibers in the cervical ECM is thought to provide scaffolding which contributes to the rigidity of the cervix, while SMCs play a supporting role by secreting ECM contents^33^. It was suggested that degradation of cervical ECM with advanced gestation allows the cervix to become a more compliant structure^34,35^. Recent literature however, has shown that the cervix is a specialized organ and its transformation during pregnancy is a complex molecular process^20,36,37^.

Despite the fact that the two sub-regions of the cervix exhibit seemingly different functions, it was assumed until recently that cervical microstructure is homogenous^30^. The latest studies employing advanced imaging techniques, i.e., MRI, optical coherence tomography (OCT), and second harmonic generation (SHG) optical analysis, have shown that the proximal and distal regions of human non-pregnant cervix are different, with the architecture of the internal os/endocervix being similar to that of the uterus itself^20,38–42^. Additionally, it was found that non-pregnant cervical stroma contains zones with fibers organized in specific orientations, either circumferential or longitudinal^20^, which may function to offset the increasing intra-uterine pressure during gestation and cause cervical shortening and dilation with the onset of labor. Thus, the cervix, once thought to be a homogenous passive structure, appears to have distinct features (i.e. a sphincter-like structure, specific functional zones, etc.) that help to maintain its important functions.

Currently, it is not clear how pregnancy affects the molecular and structural composition of different cervical zones, and what causes the modification of its morphology towards the end of gestation. We hypothesize that the cervix is a dynamic structure during pregnancy, and that capturing changes in the cervical stroma during late gestation will help redefine the human diagnosis of CI and thus improve PTB detection in high-risk women. Thus in this study, we aim to evaluate changes in the microstructure of different cervical sub-regions throughout gestation. Because the cervix in women and mice share many similarities (rev in^43^), we used a well-known mouse model of pregnancy and term labor. Our study had two objectives: (1) to examine changes associated with cervical ripening and impending delivery by using micro-MRI, and (2) to visualize and quantify these regional morphological and microstructural changes in the mouse cervix by traditional histology and immunohistochemistry methods. By including MRI in our study, we sought to examine whether this method can reveal features of cervical anatomy and structure.

## Results

### Micro-MRI evaluation of murine cervix

**Figure 1:**
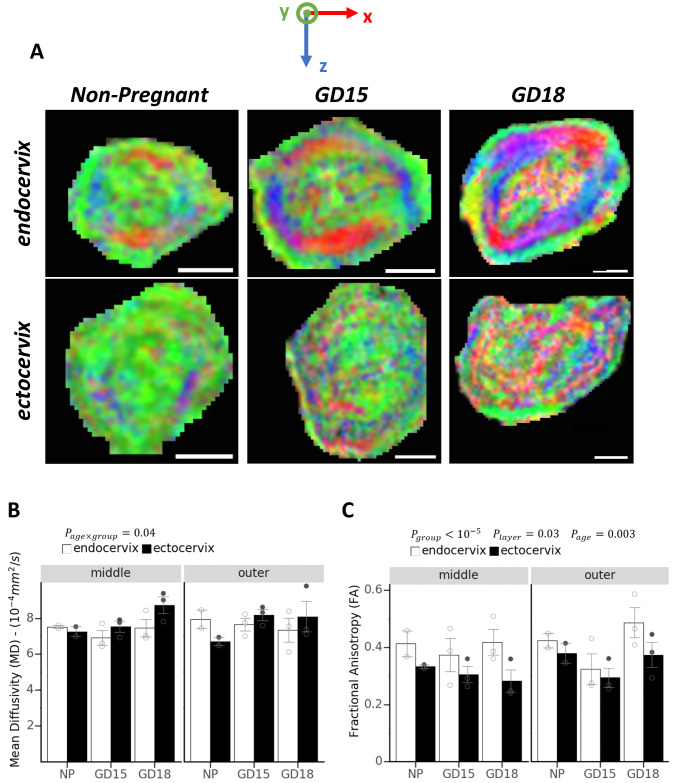
Diffusion Tensor Imaging of murine endo- and ectocervix. (**A**) Color-coded coordinate system shows the direction of restricted diffusion of water molecules in the tissue on the transverse plane. Green color or y-direction is the direction parallel to the cervix canal. Diffusion Tensor Imaging (DTI) images of endocervix (upper panel) and ectocervix (lower panel) from non-pregnant (NP), and pregnant mice (gestational day (GD) 15 and GD18). Three distinct zones are visible in the endocervix: 1) an inner longitudinal green zone of fibers around the cervical canal, surrounded by 2) circular fibers captured in red and blue, and 3) an outer longitudinal green zone. The fibers in the ectocervix are less distinct compared to the endocervix. Images were generated through FSLeyes software (FMRIB Analysis Group, University of Oxford, version 0.22.4, https://users.fmrib.ox.ac.uk/~paulmc/fsleyes/apidoc/latest/index.html). Scale bar = 1 mm. Two measured quantities from DTI images are mean diffusivity (MD) (**B**) and fractional anisotropy (FA) (**C**) and values are compared for outer and middle layers at the endocervix and ectocervix for non-pregnant and pregnant mice at two gestational time points (15GD and 18GD). Data are shown as means ± standard error of the mean (SEM). Open and gray circles represent data points related to each group, endocervix and ectocervix respectively. Main effects of group (endocervix vs ectocervix), layer (middle vs outer), or gestational age asdetermined by a mixed effect model are noted as ${P}_{group}$, ${P}_{layer}$, and ${P}_{age}$. The interaction of gestational age and cervix location is noted as ${P}_{age\times group}$. Values of the inner layer are not included in the graphs as it was not possible to separate the cervical canal value from the inner layer. A significant lower FA in the ectocervix than the endocervix shows a less organized structure at the ectocervix. Significantly higher MD in the ectocervix compared to the endocervix also represent a less dense cellular structure. Variations in diffusion parameters were also observed based on layer location and through gestational progress.

To examine mouse cervix, we applied an MRI technique called diffusion tensor imaging (DTI), which is sensitive to cellular scale restriction of water movement, and is used to infer orientation of fibers or the organization of tissue structure. Cervixes were collected from non-pregnant (NP) and pregnant (gestational day/GD15 and GD18) mice, following trans-cardiac perfusion with gadolinium-based contrast agent. Gadolinium contrast was used to enhance the contrast-to-noise ratio and decrease the acquisition time for the T2-weighted images of the fixed tissue samples. Maps of the transverse sections of murine cervix were generated from the DTI images and color coded based on the direction of least restricted diffusion (Fig. 1A). The colors red, green, and blue correspond to three axes of a coordinate system aligned to the axis of the cervical canal. We found that mouse endocervix from NP, GD15, and GD18 animals contains three well-defined zones, with specific orientation of structurally organized fibers. These zones could be described as (1) inner longitudinal zone (indicated by green), (2) a middle circular zone (blue and red), and (3) an outer longitudinal zone (indicated by green). Nearing the end of gestation (GD18) however, the inner zone has less consistent coloring compared to NP and GD15 suggesting a less directional structure. This layer structure and changes with gestational age are also evident when NP, GD15 and GD18 cervixes are examined in three dimensions from the three orthogonal views (shown in Suppl. Fig. 1). In contrast to the endocervix, the ectocervix did not show a clear pattern on the color-coded diffusion maps (Fig. 1A) and this was consistent with quantitative analysis, where the fractional anisotropy was reduced in this region (discussed below).

In addition to qualitative assessment of tissue organization based on diffusion, two quantitative metrics, mean diffusivity (MD) and fractional anisotropy (FA) are computed. Lower values of MD indicate more restriction on the diffusion of water molecules in the tissue and is reported in units of mm^2^/s. FA is a ratio ranging from 0 to 1 indicating the degree to which diffusion is restricted to a particular axis. Low values of FA, while typically not reaching zero due to measurement noise, indicate isotropic diffusion with no preferred orientation. Average MD and FA values for endo- and ectocervix layers for NP and two gestational time points (GD15 and GD18) are show in Fig. 1B,C. In computing these average values for each region, the layers for the endocervix were defined based on both the colored areas on the diffusion weighed image (DWI) (Fig. 1A) and the unweighted (b0-weighted) image (Suppl. Fig.2). Layers for the ectocervix were defined based on only the b0-weighted image (Suppl. Fig.2) as the colors on the DTI were less informative. Only values for middle and outer layers are included in the graphs and analysis. The inner layer in the MRI images comprises the cervical canal and it was infeasible to exclude voxel contaminated by canal fluid due to the coarser spatial resolution of the diffusion weighted images Fig. 1A and Suppl. Fig.2. We found that FA in the ectocervix is significantly lower than the endocervix (*p* < 10^–5^), consistent with the less organized structure described above. FA also varied between layers (*p* = 0.03) with greater anisotropy in the outer than the middle layer. There is also a statistically significant (*p* = 0.003) association between gestational age and anisotropy differences between layers, with anisotropy of the outer layer becoming more pronounced at GD18.

Analysis of MD shows a statistically significant interaction (*p* = 0.04) between gestational age (NP vs GD15 and GD18) and cervix location (ecto- or endocervix) with higher values of MD in the ectocervix at GD15 and GD18 compared to the endocervix. Increased MD indicates the loss of diffusion directionality (tissue structure that causes diffusion in one particular direction), while increased FA towards the end of gestation suggests the reorientation of the tissue fibers in a particular direction. Moreover, the value of FA for the endocervix is higher than at the ectocervix, indicating more directional fibers/structure at endocervix. Cross sectional area measurements of the outer and middle layers of endo- and ectocervix are shown in Suppl. Fig. 3. The total area of both middle and outer layer significantly increased between NP, GD15, and GD18 (*p* = 0.006), showing that the gestation factor in the model is statistically significant and independent of the layer designation. Moreover, the total area difference was more pronounced for the middle layer compared to the outer layer (*p* = 0.0001). There was also a significant difference between the total cross sectional area at ectocervix compared to endocervix (*p* = 0.03), with ectocervix having higher cross sectional area in general.

**Figure 2:**
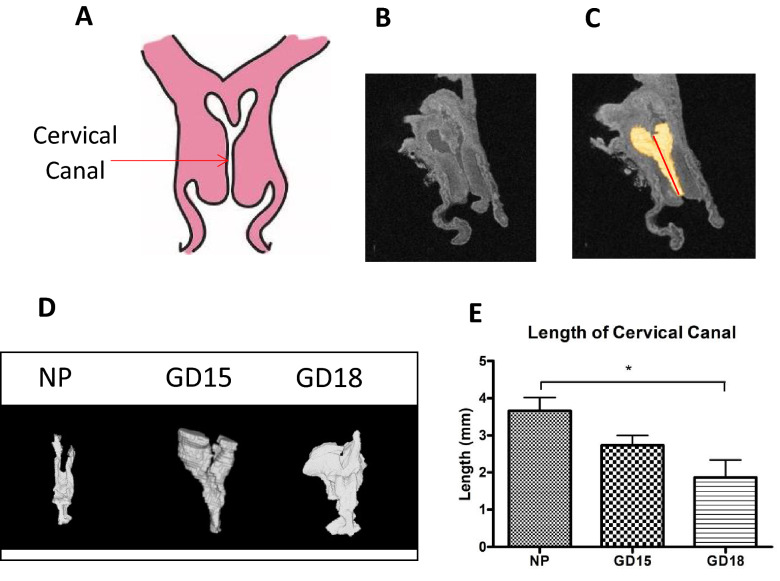
Generating 3D models of the murine cervical canal for accurate measurements of its length. (**A**) A schematic of murine cervical anatomy presented in the frontal plane, with the opening in the middle indicating the cervical canal. (**B**) A representative anatomical scan of the non-pregnant murine cervix acquired by T2-weighted imaging using a 7-T scanner (**C**) 3D models of the cervical canal (shown in yellow) were generated by the software Amira (Thermo Fisher Scientific, version 6.4.0) and were used to measure length of cervical canal. (**D**) Representative 3D models of non-pregnant (NP), and pregnant cervices on gestational day (GD)15 and GD18 show a transition from a Y shape to a U shape. (**E**) Data is presented as mean ± SEM, showing that the length of the cervical canal (measured from ectocervix to immediately before the bifurcation of the endocervix) significantly decreases at late gestation (GD18, 2.09 mm ± 0.45) as compared to NP mice (3.66 mm ± 0.35). Statistical significance was determined by one-way ANOVA with Bonferroni post-hoc test. *p < 0.05 (n = 3–6/GD).

The length of the pregnant murine cervix (mCL) was measure on T2-weighted anatomical MRI using the software Amira (Visage Imaging, version 6.4.0). 3D renderings of the cervical canal from NP and pregnant (GD15 and GD18) mice were generated for accurate length measurements, as tangential line segments following the curvature of the cervical canal were added together (Fig. 2). Our study revealed that towards the end of gestation pregnant mouse cervix changes its morphology compared to the NP cervix, as the structure loses its typical “Y” shape and takes on a “U” shape while increasing in volume (Fig. 2D). At late gestation (GD18), mCL (measured from the bottom of the ectocervix to just below the bifurcation at the endocervix) significantly decreases as compared to NP mice (2.09 ± 0.4 vs 3.66 mm ± 0.35, *p* < 0.05, Fig. 2E), similar to what is reported for CL of term pregnant women in the clinical setting.

**Figure 3:**
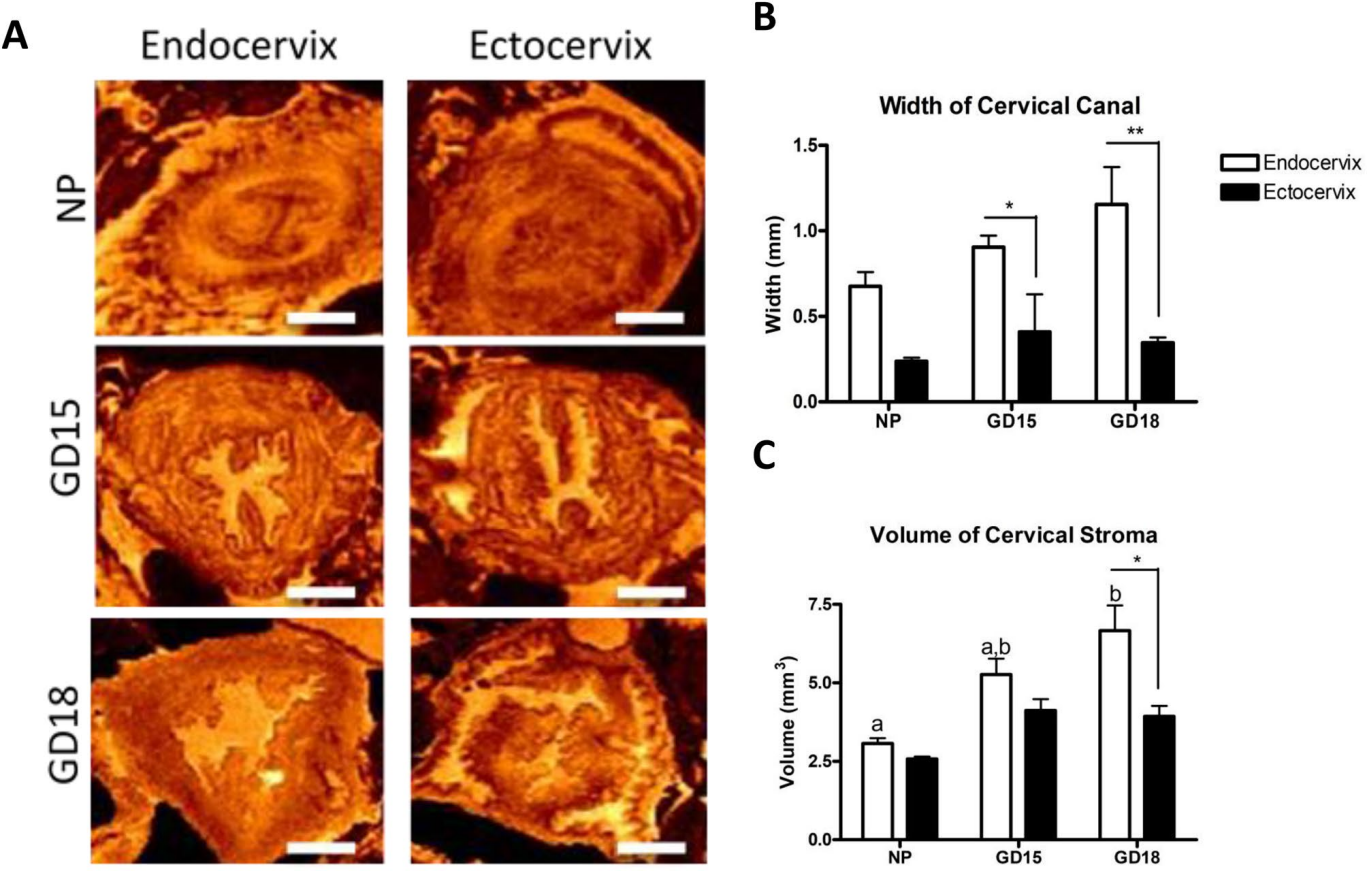
Analysis of ex-vivo T2-weighted images of the murine cervix. (**A**) The transverse plane of murine endocervix (**left**) and ectocervix (**right**) are imaged in non-pregnant (NP) and pregnant mice during mid-gestation (GD15) and late gestation (GD18), indicating that the cervical tissue and canal become wider as labor approaches. Images were generated through MNI Display (McGill Centre for Integrative Neuroscience, version 2.0.6, http://www.bic.mni.mcgill.ca/software/Display/Display.html). The orange color indicate the level of tissue hydration (brighter colors indicates more hydrated regions). Scale bar = 1 mm. (**B**),(**C**) Width and volume measurements by MNI Display (McGill Centre for Integrative Neuroscience, version 2.0.6, http://www.bic.mni.mcgill.ca/software/Display/Display.html) are shown as mean ± SEM. Results confirm that at late gestation (GD18) the canal and tissue in the region of the endocervix (**white bars**) is significantly larger than in the ectocervix (**black bars**), and these parameters are significantly different from the NP cervix. Statistical significance was determined by two-way ANOVA with Bonferroni post-test. Letters indicate significant differences in the endocervix volume across gestation. Asterisks indicate differences between the endocervix and ectocervix from the same gestational day. *p < 0.05;**p < 0.01. (n = 3–4/GD).

To take advantage of the 3D nature of MRI, volumetric analyses were performed by measuring the width and volume of the canal at the levels of the endo- and ectocervix. The detailed description of the above parameters and measurements is shown on Suppl Fig. 4. T2-weighted images of the endo- and ectocervix of NP, and GD15 and GD18 pregnant mice are shown on Fig. 3A. Visually, the cervical canal at the level of endocervix becomes wider and more hydrated (indicated by the brighter orange color) with advanced gestation, while the cervical tissue (excluding the canal) also becomes larger. The width and volume of the cervical canal and tissue were quantified in both the endo- and ectocervix (Fig. 3B,C). The width of the canal at the endocervix level increases across gestation but the difference does not reach significance, however comparisons between pregnant endo- and ectocervix show that the canal is significantly wider at the endocervix level, than the ectocervix at both, GD15 (0.9 mm vs 0.4 mm *p* < 0.05) and GD18 (1.154 mm vs 0.34 mm, *p* < 0.01) (Fig. 3B). The volume of the endocervical region at late gestation GD18 (measured by averaging of 30 consecutive slides) is significantly higher than the NP endocervix (6.6 mm^3^ vs 3.52 mm^3^, *p* < 0.01) (Fig. 3C). Furthermore, on GD18 the volume of the endocervix is significantly higher than the ectocervix (6.6 mm^3^ vs 3.9 mm^3^, *p* < 0.05).

### Histochemical evaluation of murine cervix

**Figure 4:**
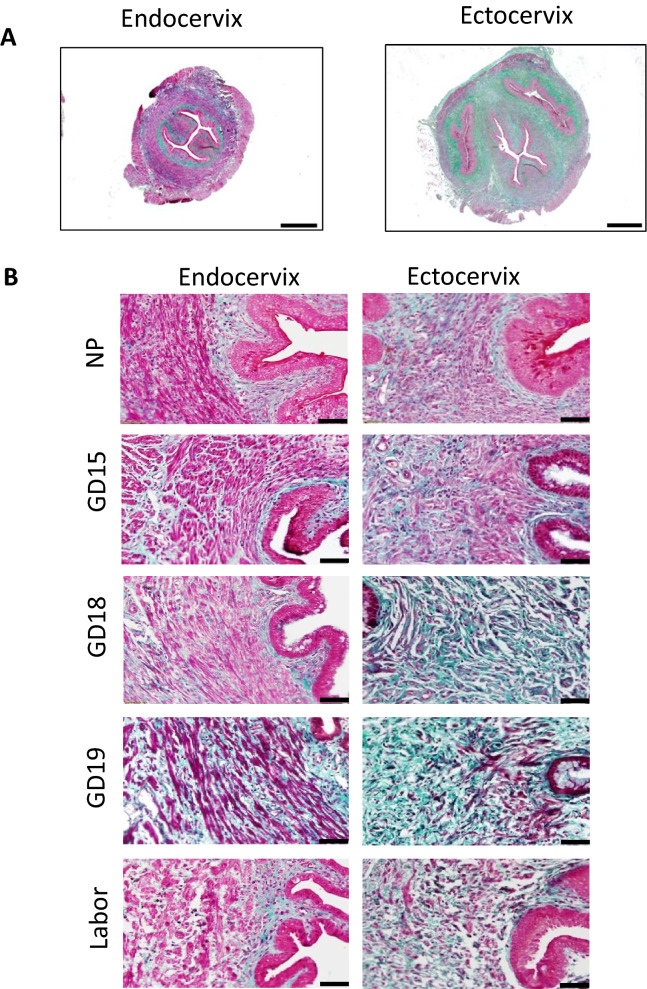
Transverse sections of histologic Masson Trichome staining of non-pregnant and pregnant mouse cervix. Masson Trichome staining was performed on formalin-fixed, paraffin-embedded sections of the endo- and ectocervix in non-pregnant (NP) and pregnant (GD15, GD18, GD19 (term not in labor), and term laboring mice (Labor) mouse cervix. Connective tissue (i.e. collagen fibers) are stained in green–blue, and cells are stained in red–purple. (**A**) Shows representative transverse sections of two regions of non-pregnant murine cervix: endocervix (**left**) and ectocervix (**right**). Magnification is at 20×. Scale bar = 500 μm. (**B**) Shows higher-powered images of the endo- and ectocervix across gestation. Magnification is at 200×. Scale bar = 50 μm.

**Figure 5:**
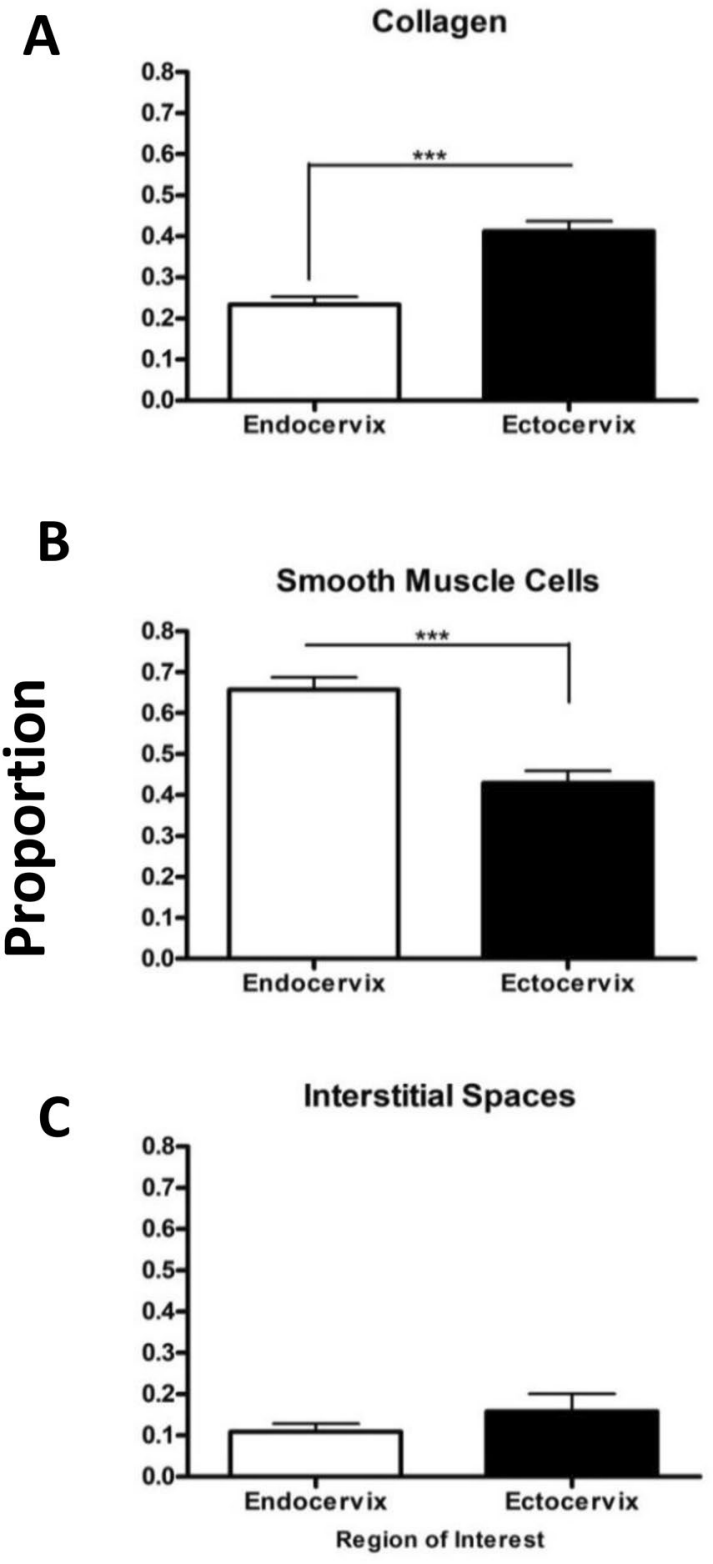
Quantification and comparison of collagen, smooth muscle cells, and interstitial spaces between the murine endo- and ectocervix by using Visiopharm Software on Masson Trichome stained cervical sections. NP, GD15, GD18, GD19, and laboring cervixes were all combined in the analysis using Visiopharm Software Engine (version 6.6.1.2572) to evaluate overall trends in the regions of interest. Data in the bar graphs are presented as mean ± SEM. Proportion of collagen (**A**), smooth muscle cells (**B**), and interstitial spaces (**C**) were calculated as the area of interest (e.g. collagen, SMCs or interstitial space) divided by the total area of the endocervix or ectocervix. Endocervix has a significantly lower amount of collagen, with a significantly higher amount of SMCs than the ectocervix. There is no significant difference in the interstitial spaces between ectocervix and endocervix (*p* = 0.31). Statistical significance was determined by t-test. *p < 0.05, **p < 0.01, ***p < 0.001. (n = 15/region).

**Figure 6:**
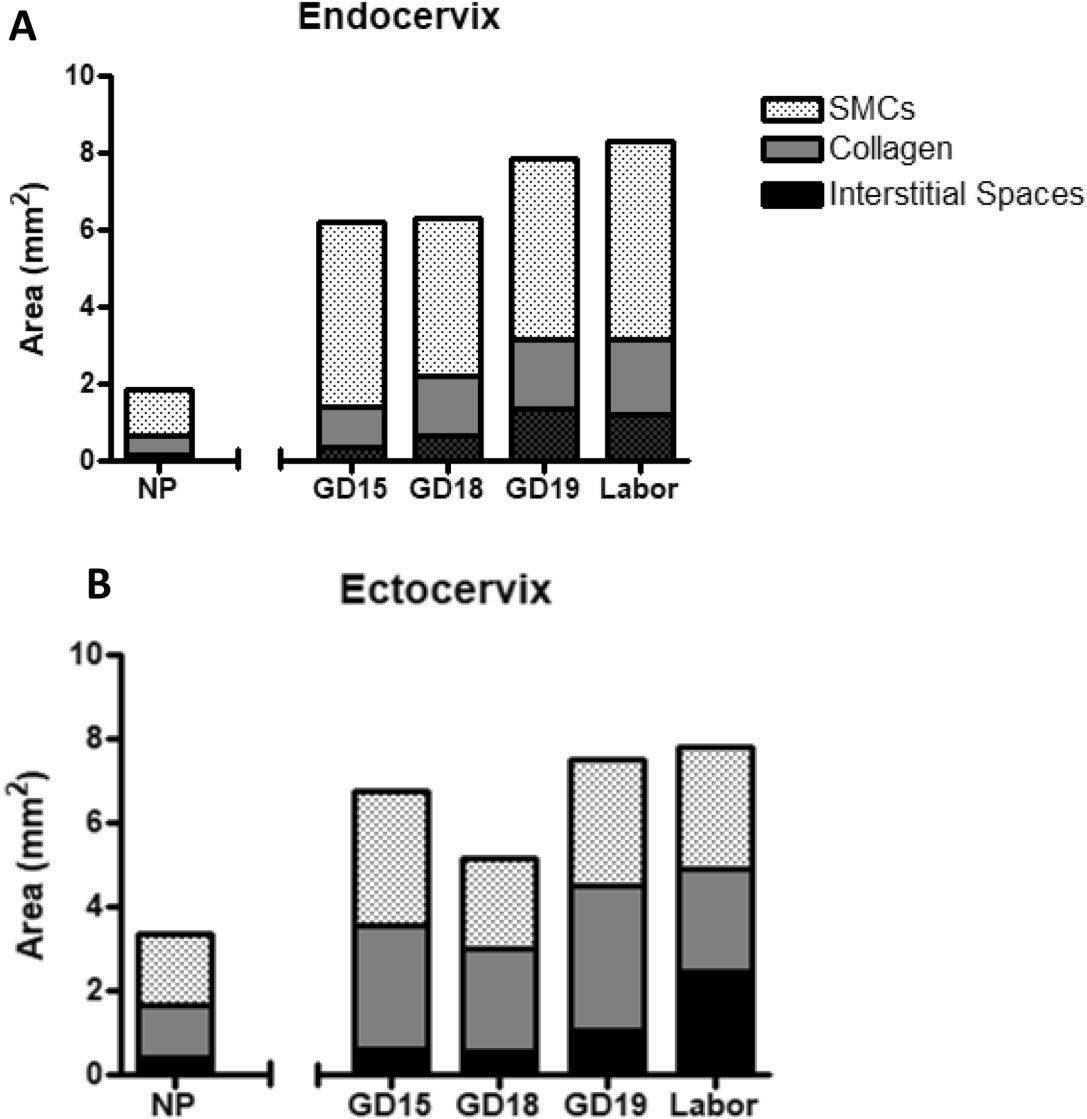
Quantification of the smooth muscle area (SMCs), collagen, and interstitial spaces in the murine endo- and ectocervix across normal gestation and term labor by using Visiopharm Software on Masson Trichome stained cervical sections. Cervixes from gestational day (GD) 15, 18, 19 and term labor, were stained by Masson Trichome histologic stain, then underwent image analysis using Visiopharm Software Engine (version 6.6.1.2572). Data is represented as stacked bars, where the length of each portion of the bar (SMCs = **dotted,** collagen = **grey**, and interstitial spaces = **black**) corresponds to their respective area (mm^2^) that contributes to entire area of the (**A**) endo- or (**B**) ectocervix at that gestational time point (i.e. the entire length of the bar is the total area of the endo-or ectocervix). Increasing areas of interstitial spaces drive the increase in total cervical area of both sub-regions of mouse cervix. (n = 3–4/GD).

To confirm zonal heterogeneity of murine endo- and ectocervix, transverse cervical sections were stained with Masson Trichome histologic staining, which marks muscle fibers in red and collagen in blue. The predominance of red staining in NP endocervix indicates a higher presence of cells, while NP ectocervix had a higher presence of collagen (Fig. 4A). High-powered images of mouse cervix across gestation indicates a major difference in the collagenous and cellular components of the endocervix, as compared to the ectocervix (Fig. 4B). At labor, the tissue of the endocervix becomes less compact, with increased presence of spaces between ECM fibers (“interstitial spaces”). The overall stereological quantification of collagen (Fig. 5A), SMCs (Fig. 5B), and interstitial spaces (Fig. 5C) in mouse cervix (including NP, pregnant and laboring samples) showed (1) a significantly lower proportion of collagen in the endocervix compared to the ectocervix (0.22 vs 0.41, *p* < 0.001); (2) with the area occupied by cells being significantly higher in the endocervix compared to the ectocervix (0.65 vs 0.42, *p* < 0.001); (3) while the increase in interstitial spaces was non-significant (0.11 vs 0.16, *p* = 0.31, NS). We further calculated the proportion of cells, collagen and interstitial spaces in pregnant mouse endo- and ectocervix throughout gestation (Fig. 6A,B). While the total area of the endo- and ectocervix increased with advanced gestation, the proportions of each component of cervical stroma (i.e. cells, collagen, and interstitial spaces) show their contribution to the total tissue area (Suppl. Table 1). For instance, in the endocervix the proportion of cells is high at GD15 (0.77) and decreases significantly at GD19 and during labor (0.59 and 0.61, *p* < 0.05 for both, compared to GD15) (Suppl. Table 1A). On the other hand, the portion of collagen and interstitial space is low at GD15, but significantly increases at term (0.16 vs 0.23, 0.06 vs 0.21, correspondingly, *p* < 0.05 for both) (Suppl. Table 1A). Interestingly, in the ectocervix, the proportion of cells is higher during late gestation (GD15 to GD19), and decreases significantly only during active labor (0.48–0.40 vs 0.37, *p* < 0.05). Similarly, the proportion of collagen decreases significantly during labor (0.32, *p* < 0.05) compared to GD18 and GD19. As a result of these changes, the relative area of interstitial spaces in the ectocervix increases dramatically during labor compared to GD15 (0.46 vs 0.10, *p* < 0.05) (Suppl. Table 1B).

**Figure 7:**
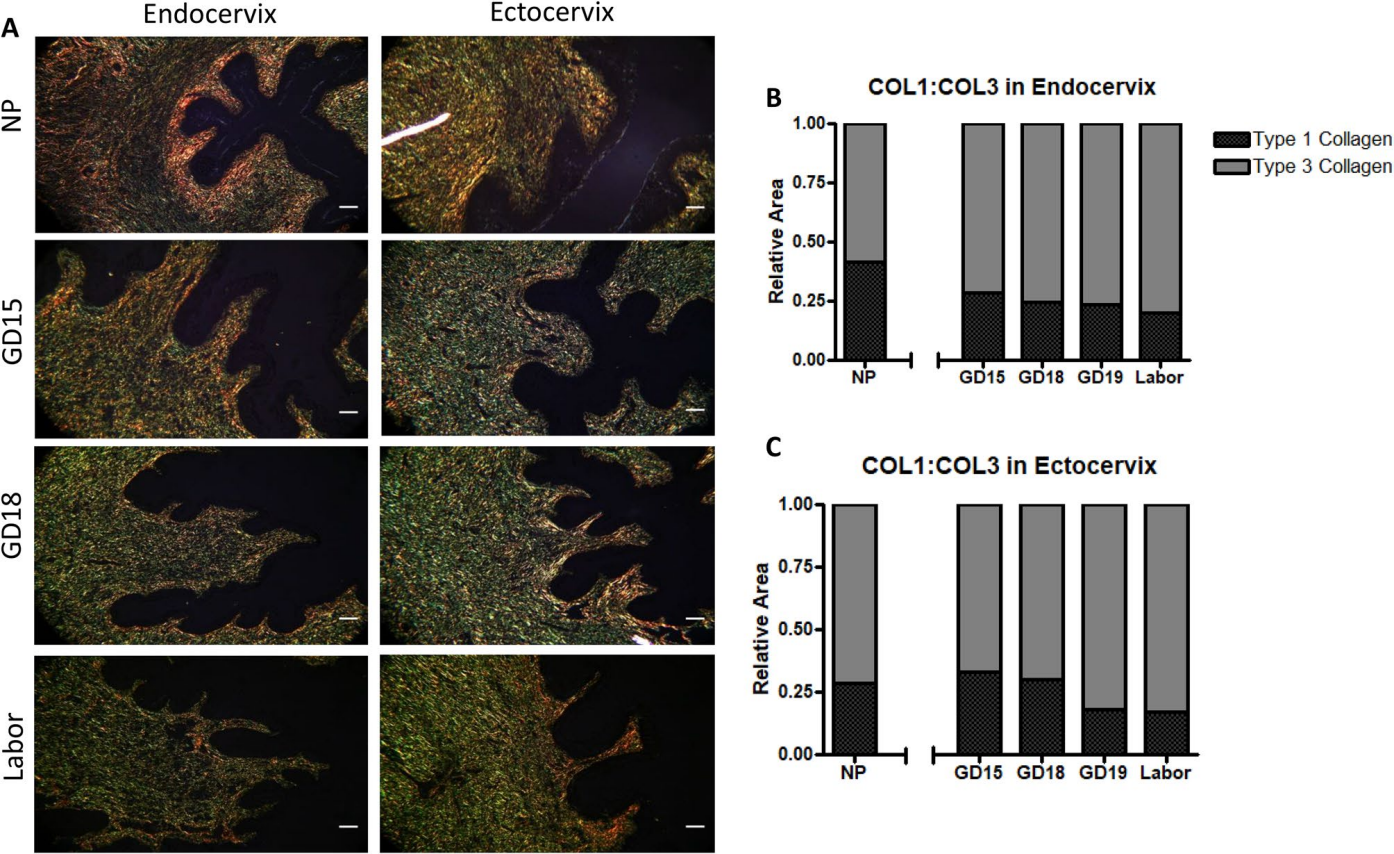
Representative transverse images of murine endo- and ectocervix across gestation treated with Picrosirius Red staining, and quantification using Visiopharm software. **(A)** Sections from NP, pregnant (gestational day (GD)15, GD18) and laboring mice were stained and images were taken under polarized light. Collagen type 1 (COL1) stains in red, and collagen type 3 (COL3)—in green. Magnification is at 200×. Scale bar = 50 μm Endocervical sections (**B**) and ectocervical sections (**C**) then underwent image analysis using Visiopharm Software Engine (version 6.6.1.2572), where the data is represented in stacked bars for each GD, as the length of each portion represents the relative area of COL1 (**black**) and COL3 (**grey**), and the sums of their relative area is equal to 1. (n = 3–4/GD).

Picrosirius Red staining was applied to visualize the different types of collagen in the mouse cervix under polarized light, where collagen type 1 (COL1), responsible for providing tensile strength to the ECM is stained in red, while collagen type 3 (COL3), contributing to tissue elasticity and extensibility, is stained in green^44,45^ (Fig. 7A). In the endocervix, COL1 occupied the inner sub-epithelial region, while the deeper sub-epithelial region is positive for COL3 fibers (Fig. 7A). While visual observation show decreased COL1 staining and increased presence of COL3 with advanced gestation, quantification of the collagen types by stereology found no significant differences (Fig. 7B,C). Throughout gestation, the ectocervix has a constant ratio of COL1:COL3 fibers, with increase in COL3 at term labor.

**Figure 8:**
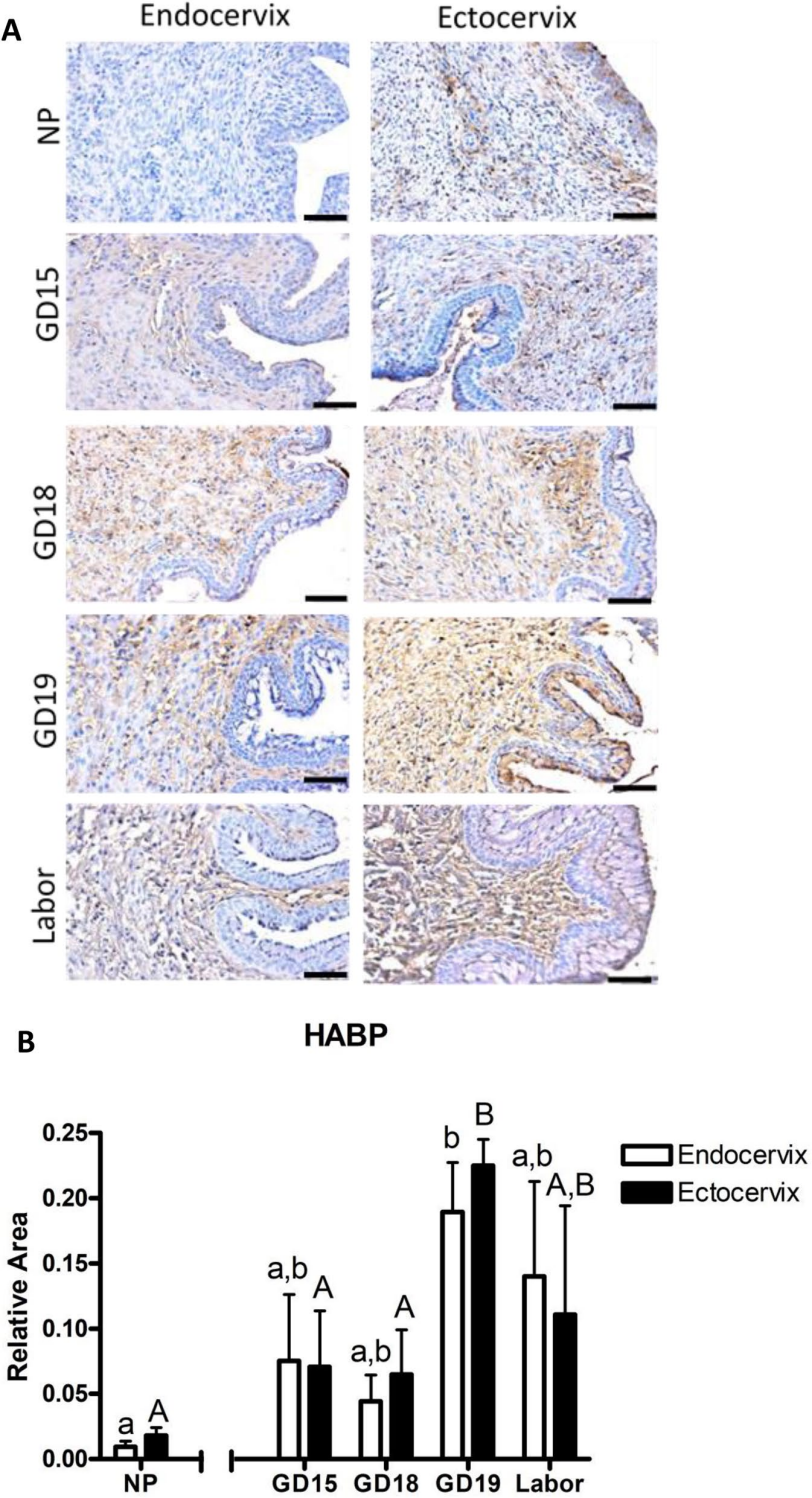
Representative images and quantification of hyaluronic acid binding protein (HABP) immunostaining in the murine endo- an ectocervix across gestation and term labor. (**A**) Shown are cross-sections of endocervix (**left**) and ectocervix (**right**) from non-pregnant (NP), pregnant (GD15, GD18, GD19), and laboring mice immunostained with antibody against HABP. Positive brown staining indicates presence of HABP. Blue shows hematoxylin counterstaining. Magnification is at 200×. Scale bar = 100 μm. (**B**) Quantification of HABP staining in the mouse cervix across gestation by image analysis using Visiopharm Software Engine (version 6.6.1.2572). Murine endocervixes (**white bars**) and ectocervixes (**black bars**). Data are presented as mean ± SEM. Statistical significance was determined by two-way ANOVA followed by Bonferroni post-test. Different lower case letters indicate significant differences in the endocervix across gestation, while different upper case letters indicate significant differences in the ectocervix across gestation. (n = 3–4/GD).

Next, transverse sections of the murine endo- and ectocervix at various gestational days were stained with antibody against Hyaluronic Acid Binding protein (HABP, a marker for hydration/tissue disruption), and quantified by Visopharm software (Fig. 8A). HABP expression is low in a NP murine cervix, but significantly increased with advanced gestation in both the endo- and ectocervix, peaking at GD19 (0.09 vs 0.18 and 0.01 vs 0.22, correspondingly, *p* < 0.05) (Fig. 8B). There are no significant differences between HABP immunostaining in the endo- and ectocervix on specific gestational days, suggesting that murine cervix becomes almost equally hydrated in both sub-regions.

### Zonal difference between endo- and ectocervix

**Figure 9:**
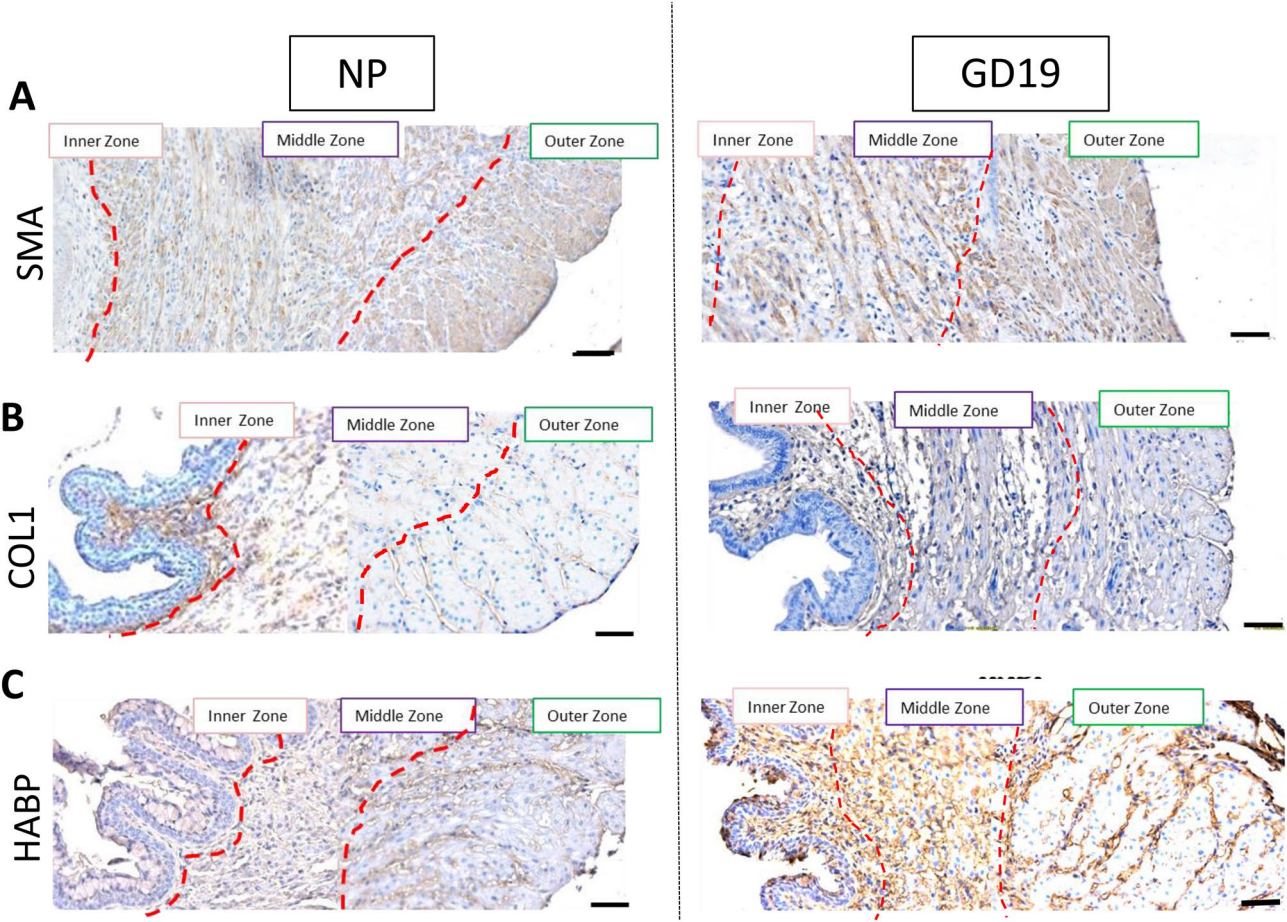
Smooth muscle actin (SMA), collagen type 1 (COL1) and hyaluronic acid binding protein (HABP) immunostaining within transverse sections of the non-pregnant and pregnant murine endocervix. Brown deposit indicate positive immunostaining. Blue shows hematoxylin counterstaining. Three zones are identified in both NP and GD19 samples: inner, middle and outer. (**A**) Staining of SMA is absent in the inner zone, while prominent in a middle zone (circular muscle fibers), and in an outer zone (longitudinal muscle fibers); (**B**) Staining of COL1 is localized in the sub-epithelial region of the inner zone specifically in the NP sample, but not in GD19 sample. (**C**) HABP shows similar weak staining throughout all three zones in NP endocervix, but the immunostaining is more intense in GD19 sample showing no heterogeneity. Magnification is at 200×. Scale bar = 100 μm.

Next, using high-powered immunohistochemistry images of transverse sections stained with antibodies against alpha-Smooth Muscle Actin (SMA), COL1, and HABP, we examined NP and term pregnant (GD19) murine endocervix. Three different zones were visually identified in both, NP and pregnant cervixes, based on heterogeneous immunostaining: inner, middle and outer (Fig. 9). SMA is absent in the inner sub-epithelial zone, whereas the middle and outer zone possess SMA-positive muscle fibers oriented in a circular and longitudinal fashion, respectively. On the other hand, the inner zone of the NP cervix shows intense COL1 immunostaining, with lower intensity in the middle and outer zone; while at term, COL1 expression in all three zones is weak. In the NP murine endocervix, HABP is weakly present throughout tissue, while term pregnant cervix (GD19) has strong HABP immunostaining in all three zones (Fig. 9).

To quantify the heterogeneity of murine endocervix, we used Visopharm software and analyzed Masson Trichrome staining of all cervixes collected throughout gestation with simultaneous quantification of three markers of interest (collagen, SMCs, and interstitial spaces, a proxy marker for HABP). Our results show that the relative area of collagen and SMCs are significantly different across the three zones described above (Table 1), indicating that the endocervix possesses specialized structures to ensure the maintenance of pregnancy.

**Table 1:** Heterogeneity of the 3 zones in the murine endocervix using all gestational samples.

Relative Area (content of interest/total area of endocervix)	Inner zone (I)	Middle zone (M)	Outer zone (O)	*p* value
Collagen	0.49	0.26	0.15	< 0.001 for **I** vs **M** and **I** vs **O**, < 0.01 for **M** vs **O**
Smooth muscle cells	0.38	0.64	0.77	< 0.001 for **I** vs **M** and **I** vs **O**, < 0.01 for **M** vs **O**
Interstitial spaces	0.13	0.11	0.10	NS for all comparisons

*NS* Non-significant.

*p* value determined by one-way ANOVA.

## Discussion

The combination of histology, immunohistochemistry, and MRI has revealed that the murine cervix possesses unique features during pregnancy. Firstly, our results show regional differences, which clearly distinguish the upper muscle-enriched compartment of the murine cervix (endocervix) from the collagenous lower region (ectocervix). Secondly, the murine endocervix is a heterogeneous structure possessing three different zones: (1) inner longitudinal collagenous zone, (2) large middle circular muscular zone (perhaps contributing to its sphincter-like property), and (3) outer muscular longitudinal zone. Thirdly, the structural properties of these cervical components change throughout gestation, creating a hydrated distensible compliant tissue that is capable of dilation during the childbirth. We concluded that anatomical and diffusion-weighted MRI has the capacity to provide insight into the microstructure of the cervix, and is a promising tool for the detection of incoming delivery that may help predict PTB in high-risk women.

Our results indicate that mouse endocervix possess a higher amount of SMCs (about 60–80% vs 30–40%) and lower amount of collagen (20% vs 40%) compared to ectocervix, which is in agreement with data recorded on hysterectomized cervices from non-pregnant women^20,46^. Vink et al.^46^ examined muscle and collagen components of human cervix using Movat Pentachrome histologic stain (similar to Masson Trichome staining) and immunohistochemistry, and found that the area of human internal os contains 50–60% of SMCs, while the external os contains 10% of SMCs^20,46^. While these important studies used NP human cervix, we aim to understand how pregnancy affects cervical microstructure. Our findings support recent literature describing cervix as a dynamic structure. We discovered that with the approach of term labor (GD19), in both cervical regions (endo- and ectocervix), area of smooth muscle is decreasing, while interstitial spaces and tissue hydration (detected by HABP) is increasing; indicating specific changes in cervical microstructure prior to labor onset, in preparation for cervical dilation during delivery. This finding supports a report by Yellon^46^ which illustrates that the cervix becomes edematous and has reduced cell nuclei density/area towards the end of gestation due to cellular hypertrophy, collagenolysis, and increased hydration^47^. Surprisingly, Picrosirius Red staining did not detect any significant difference in the collagen content of the endocervix towards labor, which might suggest that there was a change in the proportion of different collagen types: an increase in the more flexible collagen type 3 fiber with a proportional decrease in the tense collagen type 1 fibers. The results of our image analysis corroborates studies that utilize biochemical assays to examine a mechanism behind the distensible cervix. For instance, the decrease in collagen type 1 fibers has been observed in other studies, where the solubility of collagen type 1 was used as a proxy marker of total collagen content within the human cervix^11,32,37,48^. Also, Ito et al. investigated the turnover of collagen from its insoluble mature form, to a more soluble immature degradable form, thus providing the basis for the weakened structure observed in our imaging^50^. Importantly, the MRI observations show the organized layers structure still evident at GD18 with particularly anisotropic water diffusion observed in the outer layer, while when term labor approaches (GD19), the immunohistology images show a loss of organized endocervical structure, providing a means for cervical ripening^13^. Altogether, our histological results suggested that close to term the endocervix becomes more hydrated, disorganized and less rigid, losing its heterogeneity, which creates a flexible structure capable to stretch and dilate, which allows the fetus to pass during forceful labor contractions.

The combination of histology and MRI in this study provides a unique opportunity to compare the microscopic and mesoscopic scale tissue structure revealed by these two techniques. Both modalities show the presence of three zones in the murine endocervix whereas MRI also showed, albeit less distinctly, this three layer structure in the ectocervix. Immunohistochemistry/histology of the endocervix showed (1) an inner zone of collagen type 1 staining localized to the sub-epithelial area, (2) a middle zone of muscle fibers organized in a circular manner, and (3) an outer zone of muscle fibers organized in a longitudinal manner along the axis of the cervix. The DTI technique of ex-vivo MRI further corroborated the presence of these three zones, as well as their orientations. The existence of a circular structure around the uterine cavity and the uterine cervix was previously confirmed in a study on fiber architecture of nonpregnant human uterus ex vivo using MRI diffusion tensor imaging (DTI)^24^. This finding is consistent with our detection of a circular middle layer at the endocervix of non-pregnant (as well as pregnant) mice. It is the circular muscular layer within the endocervix that is thought to provide an occlusion/sphincter-like function for the maintenance of pregnancy and designed to prevent premature dilation^46,51^. DTI also detects outer longitudinal structure (a muscular zone) which might prevent premature effacement/shortening, and an inner collagenous zone (defined by immunohistochemistry). The discovery of these three zones in the murine endocervix reasonably agree with other studies that investigated the structure of mouse cervix as well as the human uterine cervix. Most notably, a recent study by Nott et al. also reported that DTI detects the presence of three zones in hysterectomized cervices from non-pregnant women: an inner longitudinal, middle circular, and outer longitudinal zone in the superior part of the cervix^27^. These authors did not detect specific zones in the distal part of the cervix (analogous to the murine ectocervix). DTI-derived measurements, such as MD and FA, are also informative regarding the change of tissue restriction on the diffusion of water molecules and how the orientation of the tissue fibers changes throughout pregnancy. MRI DTI were previously used in a study on cervical microstructure in early and late human pregnancy^52^. Qi et. al. showed that the values of FA and MD differ significantly by cervical region at late gestation compared to early gestation, although the effect of gestation age on DTI-derived measurements was not consistent for different regions. Analyses in^52^ were done on the FA and MD values obtained from the 3D structure of the two cervical regions (equivalent to layers in our study). Here, we analysed FA and MD values obtained on 2D cross-sectional images at mouse endo- and ecto-cervix. Although the sample size was too small and we could not do individual comparisons among regions, timepoints, and layers, our findings indicate a significant effect of gestational age, cervical location (ecto- and endo-) and layer location (outer and middle) on the total FA, consistent with previous reports^52^. This statement does not hold entirely for the total MD value and only the interaction of gestational age and cervical location significantly affect this value. Our study takes one step further by confirming the biological components of these three zones as being either fibrous or muscular in nature (summarized in Suppl Fig. 4). Besides detecting microstructural tissue changes, MRI has the capacity to detect changes in cervical dimensions, i.e., a widening and shortening of the cervical canal were observed towards the end of gestation in mice, which is similar to what occurs in pregnant women. Therefore, we propose that application of cervical MRI during human pregnancy can yield new insight into what constitutes a weak cervix (CI) and how deficiencies in the internal os can contribute to preterm delivery of an infant. It should be noted that the use of gadolinium contrast agents is not recommended during pregnancy and not needed for visualizing the cervix in humans as adequate spatial resolution can be obtained without a contrast agent^21^. Diffusion-weighted MRI may also find application in the study of other disorders of the cervix including cervical cancer^53^.

Previously, several studies have proposed that the detection of key biochemical markers can indicate premature cervical ripening (e.g. through presence of immune cells and collagenases, or markers of tissue hydration^9,12,13,43,47^) and subsequent PTB. However, these studies mostly existed in silos, when in reality various mechanisms work together to create a ripened structure of the cervix. Our results integrate the knowledge from previous studies about cervical ripening to provide a visual representation of the biochemical changes in the cervix through imaging, i.e. detection of collagen breakdown, increased tissue hydration and fiber re-orientation, therefore providing a visual “marker” for cervical ripening.

## Limitations

Although this study presents results that build upon and supports the recent research on cervical anatomy, it contains several limitations and caveats. Firstly, the presented MRI experiments were done ex vivo, utilizing extracted fixed organs. While this aided in obtaining higher spatial resolution, tissue fixation is known to decrease water diffusivity relative to in vivo measurements^54^. Using MRI to assess what tissue structure restrict the movement of the water molecules along a particular axis is difficult. The time scale on which water diffusion is assessed is sufficient for water molecules to cross cell boundaries and encounter a variety of molecular hindrances to their movement, thus a variety of cellular features may contribute to the observed signal changes. Another limitation of MRI-based diffusion measurements is their insensitivity to changes in the collagen network in a recent study of collagen gel phantoms^55^. In this paper, the MRI findings of oriented structure were consistent with the histological; however, additional studies in which this structure is perturbed, such as in disease, are required to isolate those tissue factors that are most important in determining MRI contrast. Moreover, fixed tissue samples for MRI and histology may also have tissue shrinkage and distortion artifacts produced by fixation, embedding, sectioning and mounting. MRI experiments were done only using cervixes from NP mice and at two gestational time points (GD15 and GD18). Technical constraints did not allow us to perform MRI experiment with laboring animals, as mice primarily deliver at night. Next, in some experiments a low sample number prevented the results from become statistically significant (i.e., Picrosirus Red staining, where we detected a trend to gestation-related changes in proportion of COL). This paper uses visual representations of tissue staining to detect cervical changes during pregnancy, which can be affected by the technique used, and differences in tissue processing. The method by which we manually quantified the amount of collagen and smooth muscle in the Masson Trichome stained sections, although insightful, is not without subjectivity and needs to be thoroughly validated in future analyses. Finally, the onset of cervical ripening and subsequent delivery requires a complex cross-talk between the fetus, uterus, and cervix. For instance, uterine contractions are linked to cervical dilation and effacement by the radial pulling of the structure^56^. While our study seeks to provide a novel way of detecting cervical changes during pregnancy, the use of ex vivo cervices inadvertently led to simplifying the model. Thus, an extensive investigation of this cross-talk, as well as the immune and hormonal pathways regulating microstructural changes in lower uterine segment during term and preterm delivery warrant a further thorough investigation.

## Conclusions

Despite the listed limitations, future applications of the knowledge gained from this study are exciting. The presence of three heterogeneous zones suggests that the cervix possesses various fail-safe features to prevent premature delivery and maintain healthy pregnancy; while during term labor the timely changes of the endocervix structure contribute to dilatation and effacement of the cervix. Our data provide scientific basis for understanding CI in human, and point to structural deficiencies of the internal os as a potential factor contributing to premature ripening and PTB. Future animal MRI studies should look at cervical changes prior to preterm labor, in the hope that the reported animal results correlate to the human clinical setting, and are able to pave the way to human studies predicting PTB in high-risk women.

## Methods

### Animals

All animal experiments were approved by the Animal Care Committee of The Centre for Phenogenomics (TCP) (Animal Use Protocol #0164H). The study was carried out in compliance with the ARRIVE guidelines. Guidelines set by the Canadian Council for Animal Care were strictly followed in handling mice. Animals were housed in a pathogen-free, humidity controlled 12 h light, 12 h dark cycle facility with free access to food and water. Female CD-1 outbred mice were naturally bred; the morning of vaginal plug detection was designated as GD1. Gestation in mice on average is up to 3 weeks, where delivery (full term) under these conditions occurred during the evening of GD19 or morning of GD20.

### Experimental design

#### Term labor mouse model

Non-pregnant (NP) and pregnant female CD-1 mice were euthanized by carbon dioxide inhalation, and cervical samples were collected on GD 15, 18, 19 (term not in labor, TNIL) and, 19 or 20 (in labor). Tissue was collected at 10 a.m. on all days with the exceptions of the labor sample that were collected once the animals had delivered at least one pup. Tissue was collected from 12–16 different animals for each day of gestation for different experiments.

### Tissue collection

#### Micro-MRI

Mice (NP, GD15 and GD18) were anesthetized with a combination of Ketamine/Xylazine (150 mg/kg/10 mg/kg) via intraperitoneal injection. For cervical tissue fixation, the thoracic cavity was cut open and animals underwent transcardiac perfusion from the left ventricle through which 30 mL of phosphate-buffered saline (PBS) containing 1 μL/mL heparin (1000 USP units/mL) and 2 mM of a gadolinium-based contrast agent (ProHance, Bracco Diagnostics, Inc., NJ, USA) followed by 30 mL of 4% paraformaldehyde (PFA, Electron Microscopy Sciences) with 2 mM of ProHance^57^. After perfusion, cervical tissues were excised by making two cuts at the uterine horns and one cut at the vagina while leaving the bladder intact to orient the specimen during scanning. Cervical tissues were then incubated in the PFA (4%) and ProHance overnight at 4℃ and finally transferred to a PBS solution containing ProHance and 0.02% sodium azide for a duration of between two weeks to a month prior to imaging.

#### Histology/immunohistochemistry

Cervical tissues were excised by carefully removing vaginal and uterine tissues to extract the cervix proper. Briefly, samples were incubated overnight in fixative (10% formalin or 4% PFA) at 4℃ and then transferred to a PBS solution after which they were promptly processed in baths with increasing concentrations of ethanol (50%, 75%, 80%, 85%, 90%, 95%, 100% ×3) for 30 min each, cleared in xylene for 30 min twice, and then embedded in wax by inserting samples into 3 baths of paraffin wax for 30 min each.

Fixed cervical tissues samples were embedded in paraffin, sectioned at 5 µm in the transverse plane and collected on Superfrost Plus slides (Fisher Scientific Ltd.). To differentiate the endocervix from the isthmus the tissue sample was sectioned along the axis of the cervical canal. The position of the endocervix was established for histological and MRI analysis based on the morphology of the cervical canal seen in cross-section. Specifically, the endocervix was measured in a section immediately preceding the opening of the cervical canal into the uterus as the region just before the cervical canal became divided into two separate canals (entering the bifurcated uterus at this point), which is illustrated in Supplemental Fig. 6.

### Masson trichome staining

Staining was performed by TCP histology laboratory. Sections were baked overnight, deparaffinised, and rehydrated in xylene, 100%, 95%, 80%, and 70% ethanol baths for 5 min each and quenched with 3% H_2_O_2_ in methanol for 20 min. Next, the tissue sections were washed and stained with a modified Masson’s trichrome histological stain. Verhoff’s Hematoxylin stain was used to stain elastic fibers and nuclei black (6 min of incubation), while Biebrich scarlet-acid fuchsin, with subsequent processing with phosphomolybdic/phosphotungstic acid and aniline blue, was used to stain collagen greenish blue (2–3 min of incubation) and cells in red. The slides were dehydrated following the staining and mounted with Surgipath Micromount mounting media (Leica Microsystems Inc.).

### Picrosirius red staining

Formalin-fixed and paraffin embedded specimens were sectioned at 5 μm in the transverse plane. Staining was performed by TCP histology laboratory. Deparaffinization of the sections began with 3 washes of xylene and then 100% ethanol, followed by 10 dips in 95% ethanol and then finally water. Celestine blue and Harris’ haematoxylin was used to stain the nuclei for 5 min each, with washes in between each stain. The slides were then dipped in acid alcohol 5 times. After washing in water, blue in Scott’s tapwater was applied for 1 min followed by another wash. To visualize collagen, the slides were stained with Picrosirius Red for 30 min and then blotted dry. The slides (n = 3–4/GD) were then dehydrated 4 times in 100% ethanol and then 3 times in xylene, and coverslipped and mounted using Permount. This protocol was adapted from Junqueira et al.^45^.

### Immunohistochemistry

Whole cervical tissue fixed in 10% of formalin or 4% of PFA was paraffin-embedded, sectioned and processed as described above. The slides were processed as described in^58^: baked overnight at 37 °C, deparaffinized and rehydrated in xylene, 100%, 95%, 90% and 70% ethanol baths for 5 min each, then quenched with 3% H_2_O_2_ in methanol for 20 min. Localization of collagen I (COL1, Abcam, AB34710), smooth muscle actin (SMA, DAKO, M0851), and hyaluronic acid binding protein (HABP, Seikagaku, 400763). Heat-induced antigen retrieval using sodium citrate were used. Briefly, slides were microwaved for 5 min at power level 6, placed on ice to cool for 20 min, and then repeatedly microwaved at power level 3. Next the slides were washed 3 times for 5 min in PBS, the individual tissue sections were selected using a wax pen (DAKO, S200230-2). Slides were blocked for one hour at room temperature using a protein blocking solution (DAKO, X0909). Sections were then incubated with primary antibodies for COL1 (1:500), SMA (1:100), and HABP (1:100) overnight at 4 °C. On the next day, sections were washed 3 times in PBS and then incubated with a biotinylated secondary anti-rabbit or anti-mouse antibody (1:300; Vector Laboratories Inc.)^59^. Rabbit immunoglobulin G (IgG) was used as the negative control at the same concentration as the primary antibodies (Suppl. Fig. 5). Finally, sections were treated with streptavidin–horseradish peroxidase solution (1:2000; DAKO), then developed with a 3,30-diaminobenzidine (DAB) kit (Vector Laboratories Inc.). Slides were counterstained with Hematoxylin Solution (Sigma-Aldrich) and mounted with Surgipath Micromount mounting media (Leica Microsystems Inc.)^59^.

### Magnetic resonance imaging

A multi-channel 7.0 T MRI with a 40 cm diameter bore magnet (Varian Inc. Palo Alto, CA) and outfitted with a custom-built 16-coil solenoid array was used to image 16 samples concurrently^60^. T2-weighted 3D images of the whole murine cervix were acquired using a cylindrical k-space acquisition with the following parameters: repetition time (TR) of 350 ms, and echo time (TE) of 12 ms per echo for 6 echoes, four averages, field-of-view (FOV) of 20 × 20 × 25 mm^3^ and matrix size = 504 × 504 × 630 giving an image with 0.040 mm isotropic voxels^51^. Scan time was 14 h. The raw data was reconstructed using in-house developed software and corrected for geometrics distortion as described in^61^. To aid in subsequent analysis, a single 3D image was selected as a “standard” image of the murine cervix and all other images were reoriented and resampled into this standard orientation. This alignment used rigid-body transformations determined from manually placed landmarks placed using the MNI register software (Montreal Neurological Institute, Canada). Visualization and segmentation of the murine cervix was performed in the MNI Display software (Montreal Neurological Institute), and 3D rendering of the cervical canal was performed by using the software Amira (Visage Imaging, version 6.4.0).

Diffusion weighted MRI data was acquired with the following parameters: fast spin echo readout, echo train length of 6, with a TR of 350 ms, first TE of 30 ms, and a TE of 6 ms for the remaining 5 echoes, two averages, field-of-view 14 × 14 × 25 mm^3^ and a matrix size of 180 × 180 × 324 yielding an image with 0.078 mm isotropic voxels (3D units of space). Five b = 0 s/mm^2^ images and 30 high b-value images (b = 2147 s/mm^2^) in 30 different directions were acquired. The utilization of 30 directions is based on the “Jones 30 Scheme”^62^ and allowed for calculation of a diffusion tensor, summarizing both the degree and direction of diffusion at each image location (DTI).

Raw diffusion weighted data was reconstructed and corrected for spatial distortion as described above and then processed for diffusion tensor analysis using the FSL suite of tools (FMRIB Analysis Group, University of Oxford). This included computation of mean diffusivity (MD), fractional anisotropy (FA), and axial diffusivity (diffusivity along the principle axis) for each voxel in the 3D image. As described for the T2-weighted images, the diffusion images were reoriented to a common coordinate system such that the axis of the cervical canal was aligned with the y-axis and the corresponding rotations were applied to the axes of the diffusion tensor. This facilitated colour-coded visualization of the principle diffusion axis where the hue represented the axis orientation and luminance corresponded to the magnitude of axial diffusivity. (Fig. 1A). Color-coded maps of the murine cervix were generated by the FSLeyes software (FMRIB Analysis Group, University of Oxford). The position of the transverse sections was chosen based on the cervical anatomy. Since the MRI data is acquired in 3D with isotropic resolution, 2D sections were extracted retrospectively from the 3D data block after both b0-weighted and diffusion weighted images were reoriented, resampled and interpolated into the "standard" orientations.

### Image analysis

#### Analysis of T2-weighted images

Various dimensions and ROI in the murine cervix were measured on the T2-weighted 3D images (Suppl Fig. 6). Width and length of cervical canal were measured using the ruler function in Amira (Visage Imaging, version 6.4.0) where tangential lines following the curvature of the cervical canal were added up to a total length in millimeters (mm). The upper and lower limit of the cervix (the endocervix and ectocervix, respectively) were segmented into portions of 30 slices each using MNI Display for the purpose of computing their respective volumes^63^.

#### Analysis of DTI images

Two cross-sections of the transverse sections, namely, endocervix and ectocervix, are selected for further analysis based on appearance properties. Masks were created to separate different layers, namely inner and middle, on both endo- and ectocervix by manually painting regions of interest. To create masks on endocervix, both the diffusion weighted image (DWI) (Fig. 1A) and non-diffusion weighted (b0-weighted) image (Suppl. Fig. 5) were used. Layer masks for the ectocervix were defined based on only the b0-weighted image (Suppl. Fig. 5) as the colors on the DTI were less informative. Average of mean diffusivity (MD) and fractional anisotropy (FA) values of pixels from the defined masks were then computed. Total area for each layer was computed by multiplying the total number of pixels by the area of one pixel (0.078 * 0.078 mm^2^).

#### Analysis of tissue staining

All slides were scanned (Axio Scan.Z1 Slide Scanner, Carl Zeiss Microscopy) and the pictures were imported into Visiopharm NewCast Software (version 6.6.1.2572) Engine and Viewer software module for quantification. Different tissue sections from either the endocervix or ectocerivx were labeled and sampled (region of interest/ROI: 10–30% of total region area depending on the section size in order to get about 20 frames for each section) and this procedure was repeated in 3–4 samples/biological replicates. For Masson Trichome stained slides, the areas of the cervical tissue occupied by collagen, muscle cells, or interstitial spaces (pockets of white areas) were manually labelled and counted by two independent observers. The relative amount of collagen, cells, or interstitial spaces for each individual cervical tissue sample was calculated as the ratio of the area occupied by collagen, cells, or interstitial spaces relative to the total masked ROI area. For Picrosirius stained slides, red fibres and green fibres were manually labelled and counted in the masked ROI, and the relative area of collagen was calculated as area of collagen type 1 or 3 divided by the total masked ROI area. The areas of the cervical tissue that positively stained for HABP (visualized by the brown deposition), and the rest of the tissue that were stained in the Hematoxylin blue. Slides were manually colour-coded, using the K-means clustering function on the software, the rest of the cervix was color-coded appropriately. All of these quantities were compared across normal gestation and labor (n = 3–4/GD).

### Statistical analyses

Statistical analyses were performed using GraphPad Prism ( version 4.0, GraphPad Software, Inc.). For cervical gene expressions in the TL and PTL models, statistical analysis was performed using t-tests to compare mid-gestation (GD15) expression levels with gene expression during laboring samples, as well as between controls and PTB-induced treatments. When comparing ROI (i.e. endocervix, and ectocervix) across gestation, two-way ANOVA was used with Bonferroni post-hoc test. Significantly different results were indicated by different letters or by asterisks: *(*p* < 0.05), **(*p* < 0.01) and ***(*p* < 0.001).

Linear mixed model (LME) analysis was performed in R (R Core Team, 2020) with lme4^64^ to investigate the effect of pregnancy stage (NP, GD15 and GD18), cervix location (endo- or ectocervix) and layer location (middle and outer) on diffusion parameters, namely FA and MD values. An one-way analysis of variance (ANOVA) test was performed on the model to assess contribution of each factor and interaction to the total variance observed. Statistical significance was assumed for *p* < 0.05.

## Supplementary Information

Supplemental Figures.

Supplemental Table 1.
