# Supplementary material for: Combination of histochemical analyses and micro-MRI reveals regional changes of the murine cervix in preparation for labor
Source: Sci Rep. 2021 Mar 1;11:4903. doi: 10.1038/s41598-021-84036-9 (PMC7921561; doi:10.1038/s41598-021-84036-9)
Supplement: Supplementary file 1 — Supplemental Figures. [file 41598_2021_84036_MOESM1_ESM.pdf]

Combination of histochemical analyses and micro-MRI reveals regional changes of the murine cervix in preparation for labor

Antara Chatterjee[1,2], Rojan Saghian[3,4], Anna Dorogin[2], Lindsay S. Cahill[4], John G. Sled[3,4,5], Stephen Lye[1,2,5], Oksana Shynlova[1,2,5]

[1] University of Toronto, Physiology, Toronto, Canada,

[2] Sinai Health System, Lunenfeld-Tanenbaum Research Institute, Toronto, Canada

[3] University of Toronto, Medical Biophysics, Toronto, Canada

[4] Hospital for Sick Children, Mouse Imaging Centre, Toronto, Canada

[5] University of Toronto, Obstetrics and Gynecology, Toronto, Canada

Corresponding Author: Dr. Oksana Shynlova

Lunenfeld-Tanenbaum Research Institute, Sinai Health System

25 Orde Street, Room 6-1017, Toronto ON

T: (416) 586-4800 ext. 5635

Email: shynlova@lunenfeld.ca

Supplemental Figure 1

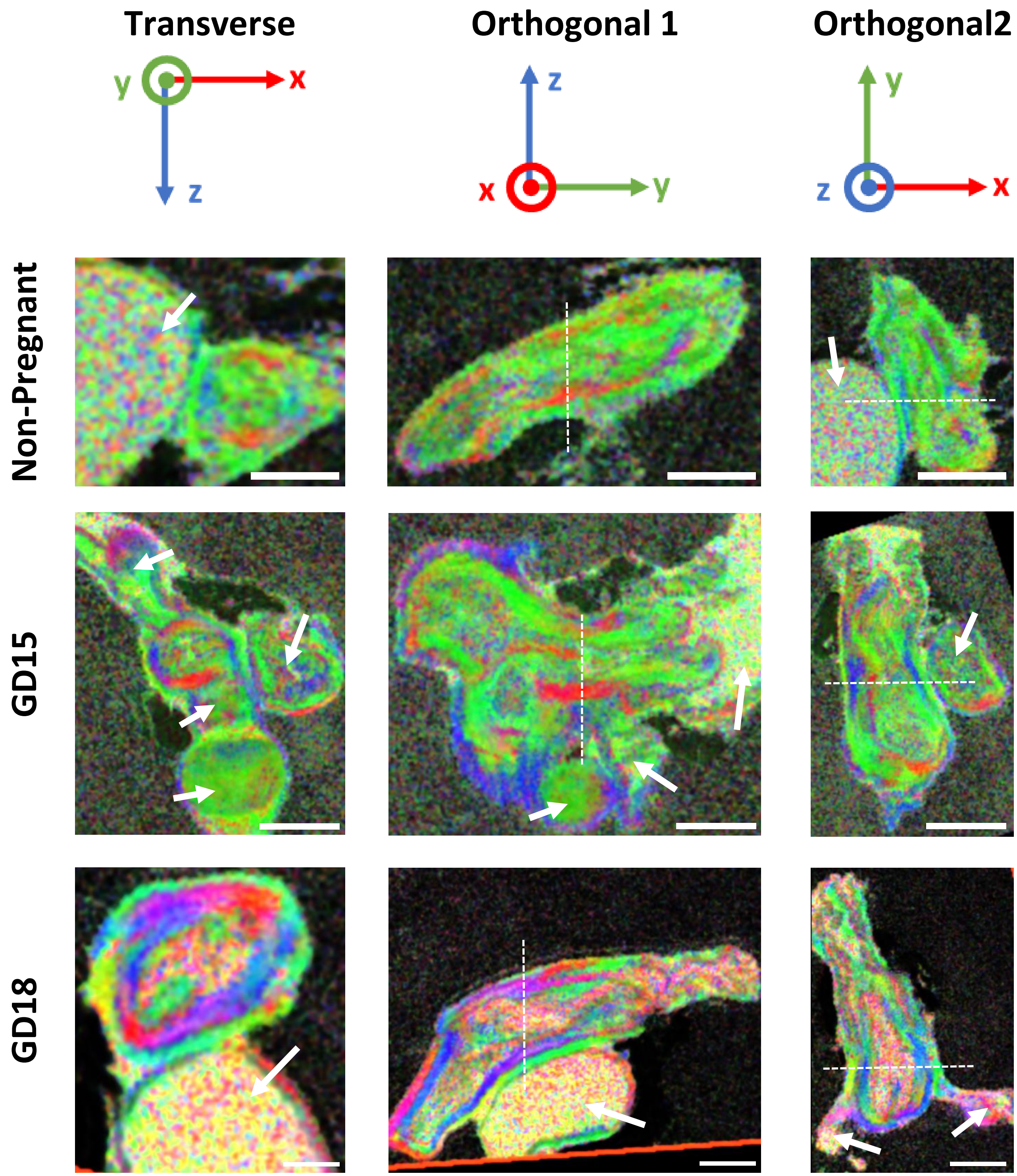

Supplemental Fig.1. Diffusion Tensor Imaging of murine endocervix from three orthogonal views. Representative Diffusion Tensor Imaging (DTI) images of endocervix from three different viewpoints: transverse and two other orthogonal views, for non-pregnant (NP) and pregnant (at 15 and 18 gestational day (GD)). Images were generated through FSLeyes software (FMRIB Analysis Group, University of Oxford, version 0.22.4, https://users.fmrib.ox.ac.uk/~paulmc/fsleyes/apidoc/latest/index.html). Color-coded coordinate systems at the top panel show the direction of restricted diffusion of water molecules in the tissue. Red indicates diffusion in parallel to the x-direction, blue indicates the water molecule movement in parallel to the z-direction, and green shows diffusion in parallel to the y-direction (which is parallel to the cervix canal). Three distinct layers are evident from the transverse and orthogonal views: i) inner, ii) middle circular, and iii) outer longitudinal layer. A green outer layer seems to become thinner by gestation. Change of color from red to blue and vice versa, on the transverse view, represent the middle circular layer. This is also evident from two orthogonal views with red and blue colors showing in-and-out of the page direction in orthogonal-1 and orthogonal-2 views respectively. Inner layer, on the other hand, seems more longitudinal for NP and at 15GD (with green color dominant at this layer) but seems to lose structural integrity at 18GD (towards the end of pregnancy). White dashed line indicates the position of the transverse plane (endocervix) at the orthogonal views. White arrows point to extra tissues and bladder that are still attached to the cervical tissue.

Supplemental Figure 2

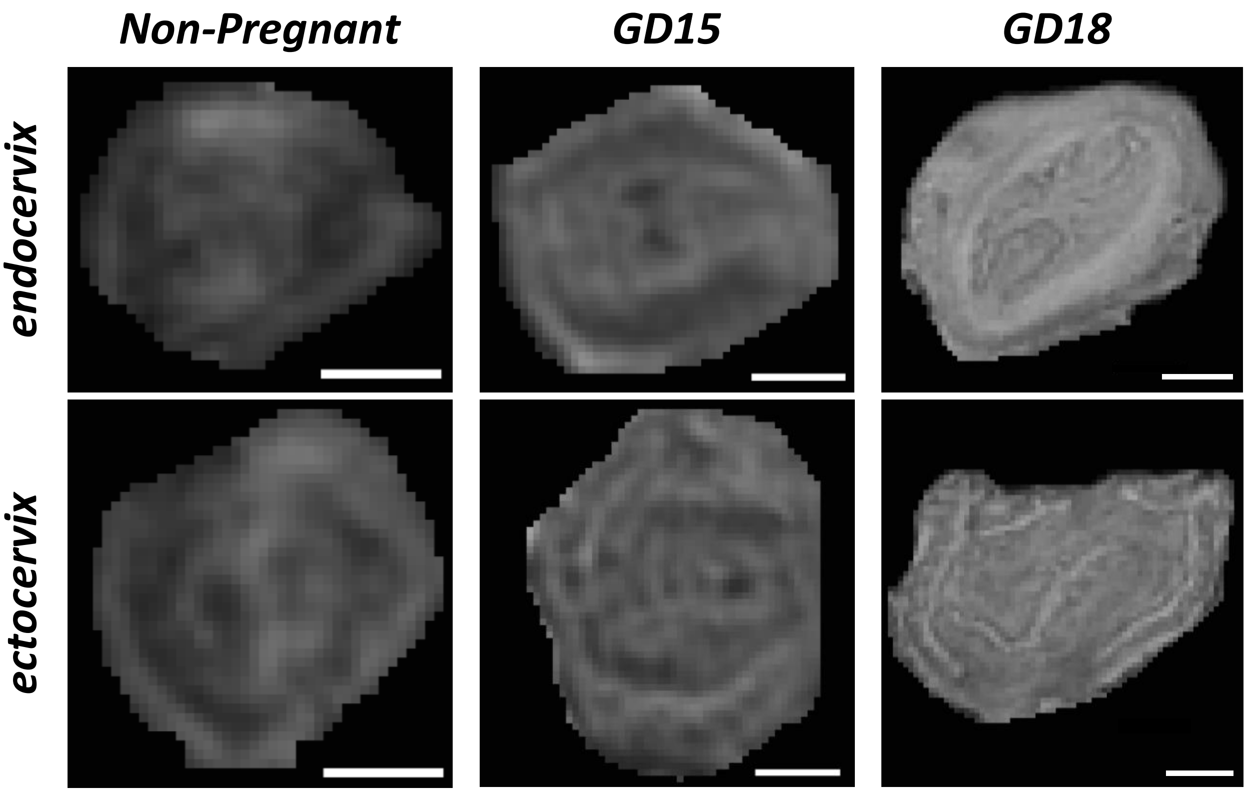

Supplemental Fig.2. B0-weighted images of murine endo- and ectocervix. Non-diffusion weighted (b0-weighted) image of endo- and ectocervix corresponding to color coded diffusion weighted images (DWI) in (Fig. 1).). Images were generated through FSLeyes software (FMRIB Analysis Group, University of Oxford, version 0.22.4, https://users.fmrib.ox.ac.uk/~paulmc/fsleyes/apidoc/latest/index.html). Layer structure in both endo- and ectocervix is distinguishable and were used as guide lines in creating layer masks (for outer and middle layers) for analysis. To create masks on endocervix, both the DWI and b0-weighted images were used. Scale bar = 1mm.s.

Supplemental Figure 3

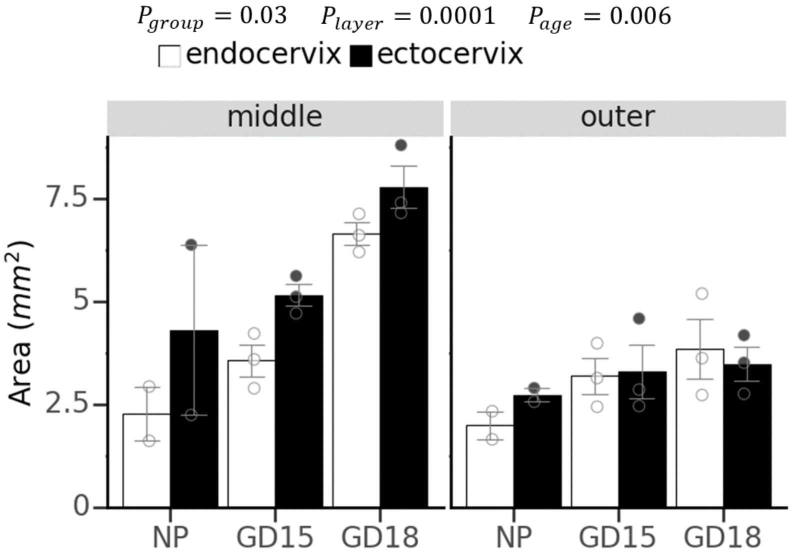

Supplemental Fig. 3. Quantification of the area of middle and outer layer at endo- and ectocervix layers of non-pregnant and pregnant mice. Area measurements of the middle and outer layer at the endocervix and ectocervix for non-pregnant (NP) and pregnant (at 15 days of gestations (GD) and 18GD). Data are shown as means ± standard error of the mean (SEM). Open and gray circles represent data points related to each group, endocervix and ectocervix respectively. Main effects of group (endocervix vs ectocervix), layer (middle vs outer), or gestational age as determined by a mixed effect model are noted as P_{group}, P_{layer}, and P_{age}. The total area of both middle and outer layer increases when gestation happen and keeps increasing during gestation. However, this increase in significantly higher for the middle layer compared to the outer layer. Total cross section area of ectocervix is also generally bigger than the total cross section area of the endocervix. Layers for the ectocervix are defined based on the b0 weighted image as the colors on the DTI were not distinctive of layers.

Supplemental Figure 4

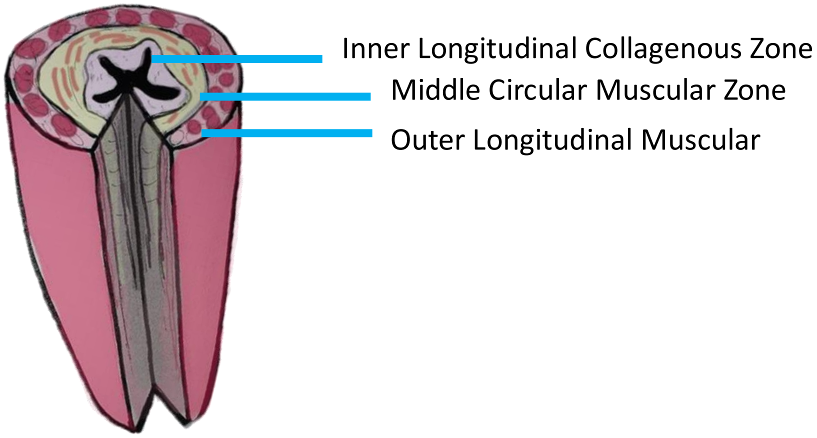

Supplemental Fig.4. Depiction of the three zones in the transverse section of the endocervix. **Depiction of murine cervix and its three layers.** Histology, immunohistochemistry and DTI discovered the presence of three layers in the endocervix: 1) inner longitudinal collagenous zone, 2) middle circular muscular zone, and 3) outer longitudinal muscular zone.

Supplemental Figure 5

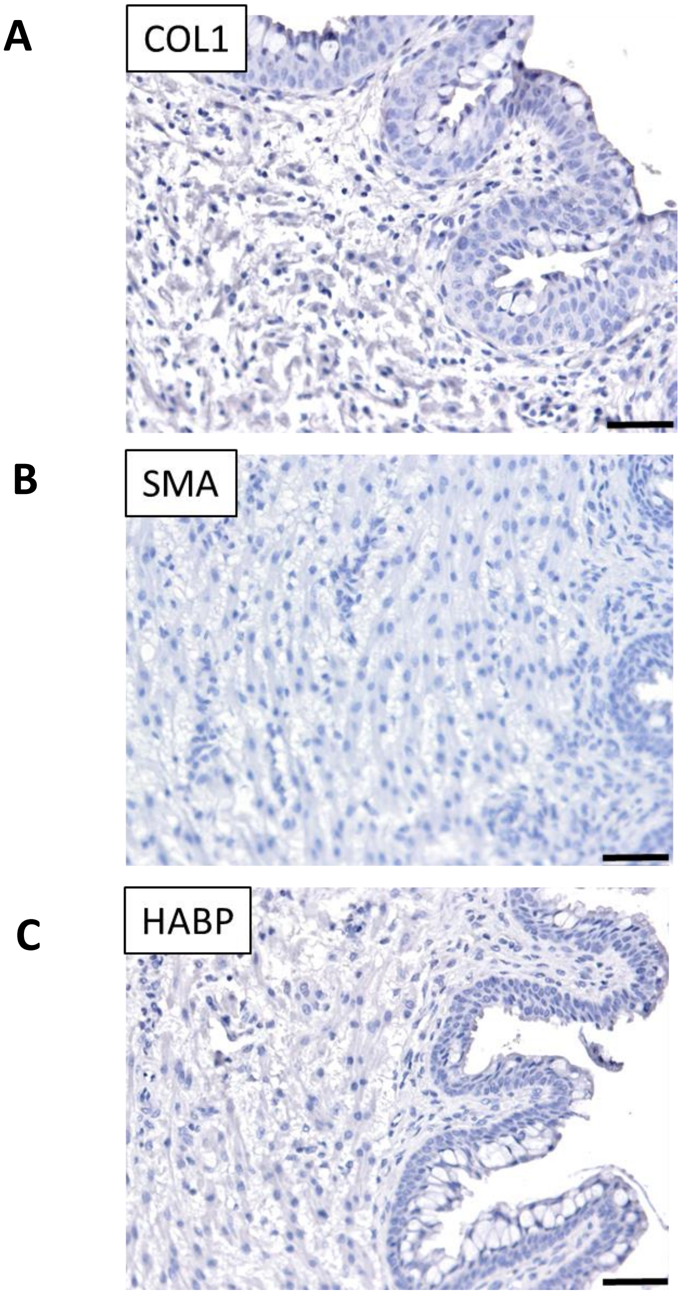

Supplemental Fig. 5. Negative controls (IgG staining) for collagen (COL1), smooth muscle actin (SMA), and hyaluronic acid binding protein (HABP) immunohistochemistry on GD19 tissue sections. Rabbit immunoglobulin G (IgG) was used as the negative control for following antibodies: (**A**) COL1, (**B**), SMA and **(C)** HABP. Magnification is at 200x. Scale bar = 100 μm**.**

Supplemental Figure 6

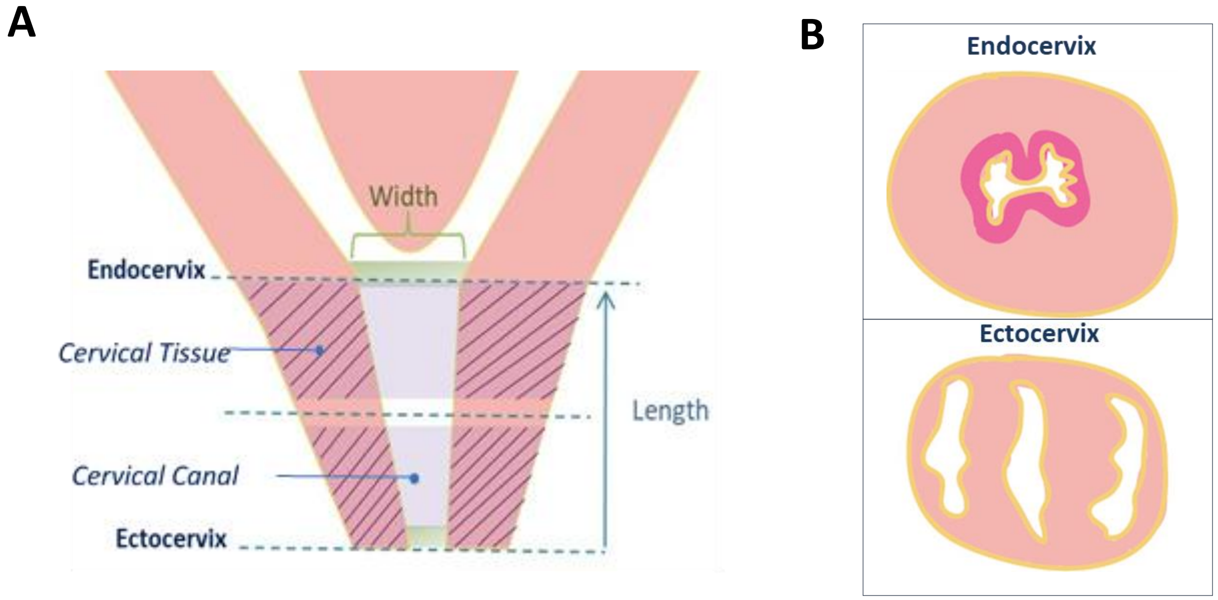

Supplemental Fig. 6. Schematic of murine cervix, its various dimensions and regions of interest (ROI). (**A**) Width measures distance across the openings at the endo- and ectocervix. Length measures the distance of the cervical canal (from the bifurcation at the endocervix to the opening at the ectocervix). The tissue surrounding the endo- and ectocervix (presented by purple lines in the schematic) was segmented digitally into 30 transverse slices by using the software MNI Display (McGill Centre for Integrative Neuroscience, version 2.0.6, http://www.bic.mni.mcgill.ca/software/Display/Display.html)) so that the volume of cervical tissue (excluding canal) could be measured. MRI was then used to determine the length, width, and volume of the cervix. (**B**) Depictions of the transverse view of murine endocervix and ectocervix - the two ROI selected for investigation of microstructural changes by MRI, histology and immunohistochemistry.
