# Supplementary material for: Combination of histochemical analyses and micro-MRI reveals regional changes of the murine cervix in preparation for labor
Source: Sci Rep. 2021 Mar 1;11:4903. doi: 10.1038/s41598-021-84036-9 (PMC7921561; doi:10.1038/s41598-021-84036-9)
Supplement: Supplementary file 2 — Supplemental Table 1. [file 41598_2021_84036_MOESM2_ESM.pdf]

<u>Manuscript Title:</u> Combination of histochemical analyses and micro-MRI reveals regional changes of the murine cervix in preparation for labor

<u>Authors:</u> Antara Chatterjee, Rojan Saghian, Anna Dorogin, Lindsay S. Cahill, John G. Sled, Stephen Lye, Oksana Shynlova

Supplemental Table 1. Proportion of cells, collagen, and interstitial spaces in endocervix (A) and ectocervix (B) during gestation and term labor (TL)

A

	GD				
Proportion	GD15	GD18	GD19	Labor	P-value*
SMCs	0.77	0.65	0.59	0.61	<0.05 for GD15 vs. GD18,GD19, and Labor
Collagen	0.16	0.24	0.23	0.23	<0.05 for GD15 vs. GD18,GD19, and Labor
Interstitial Spaces	0.06	0.12	0.21	0.17	<0.05 for GD15 vs. GD19

Proportion determined by area of marker of interest/total area of cervical sub-region studied
*p-value determined by two-way ANOVA with Bonferroni post-hoc test

B

	GD				
Proportion	GD15	GD18	GD19	Labor	P-value*
SMCs	0.48	0.42	0.40	0.37	<0.05 for GD15 vs. Labor
Collagen	0.43	0.47	0.45	0.32	<0.05 for Labor vs. GD18 and GD19
Interstitial Spaces	0.10	0.12	0.16	0.46	<0.05 for Labor vs. GD15, GD18, GD19

Proportion determined by area of marker of interest/total area of cervical sub-region studied
*p-value determined by two-way ANOVA with Bonferroni post-hoc test
